# Sulfasalazine Microparticles Targeting Macrophages for the Treatment of Inflammatory Diseases Affecting the Synovial Cavity

**DOI:** 10.3390/pharmaceutics13070951

**Published:** 2021-06-24

**Authors:** Monica-Carolina Villa-Hermosilla, Ana Fernández-Carballido, Carolina Hurtado, Emilia Barcia, Consuelo Montejo, Mario Alonso, Sofia Negro

**Affiliations:** 1 Department of Pharmaceutics and Food Technology, School of Pharmacy, Universidad Complutense de Madrid, Ciudad Universitaria s/n, 28040 Madrid, Spain; mcvhermosilla@ucm.es (M.-C.V.-H.); ebarcia@ucm.es (E.B.); marioalonsogonzalez@ucm.es (M.A.); soneal@ucm.es (S.N.); 2 Institute of Industrial Pharmacy, Universidad Complutense de Madrid, Ciudad Universitaria s/n, 28040 Madrid, Spain; 3 Department of Pharmaceutical and Health Sciences, Faculty of Pharmacy, CEU San Pablo University, 28925 Madrid, Spain; carolina.hurtadomarcos@ceu.es (C.H.); montejo@ceu.es (C.M.)

**Keywords:** microparticles, sulfasalazine, macrophages, anti-inflammatory effect, α-tocopherol, controlled release

## Abstract

Rheumatoid arthritis (RA) is a chronic inflammatory disease with sulfasalazine (SSZ) extensively used for long-term treatment of both juvenile and adult RA. Its use is associated with adverse effects and toxicity due to its non-selective biodistribution. Macrophages play an important role in inflammatory processes. In order to target SSZ to macrophages in this work two microparticulate systems (MPs) are developed: SSZ-loaded PLGA MPs without and with α-tocopherol, with particle sizes lower than 5 μm and encapsulation efficiencies of 81.07 ± 11% and 63.50 ± 6.62%, respectively. Release of SSZ from MPs prepared with α-tocopherol was prolonged for 20 days. In RAW 264.7 cell macrophages MPs prepared with α-tocopherol were captured faster. Cell viability studies confirmed that SSZ-loaded MPs prepared without and with α-tocopherol did not produce cytotoxicity at the concentrations assayed. The anti-inflammatory activity of SSZ-loaded MPs was studied by quantifying interleukins IL-1, IL-6 and TNF-α in macrophages. All formulations produced a significant reduction of cytokine concentrations after 24 and 72 h, indicating that release of SSZ from the MPs was able to inhibit the inflammatory response induced by lipopolysaccharide (LPS). Gene expression of IL-1, IL-6 and TNF-α was decreased by SSZ-loaded MPs. SSZ-loaded MPs prepared with α-tocopherol will potentially allow increasing the residence time of SSZ in the synovial cavity, prolonging its duration of action, and reducing the adverse effects associated with its non-selective biodistribution.

## 1. Introduction

Antirheumatic drugs are used in inflammatory arthritis, predominantly in rheumatoid arthritis (RA), psoriatic arthritis, ankylosing spondylitis and many other autoimmune diseases. RA presents a global prevalence of around 0.5–1% and is associated with symptoms such as pain and stiffness in multiple joints. In the early stages of the disease around one third of the patients suffer from symptoms either at one location or at a few scattered sites [1,2]. In most cases proliferation of the synovial tissue occurs with formation of synovial pannus and joint destruction due to the increased levels of inflammatory mediators. Nonsteroidal anti-inflammatory agents (NSIADs) and corticosteroids are commonly used for its treatment but have little effect in altering the natural history and outcomes of RA, which can lead to cartilage and joint destruction and severe disability. The introduction in therapeutics of the so-called DMARDs (disease-modifying anti-rheumatic drugs) has clearly improved the treatment of RA, thereby having a marked influence on the quality of life of patients suffering from the disease. Among them, sulfasalazine (SSZ) remains one of the most commonly used DMARDs in clinical practice [3,4], with many clinical guidelines recommending its use, especially in non-serious RA [5], for which weekly doses of 7.5–15 mg are indicated [6]. Sulfasalazine is also extensively used in the long-term therapy of juvenile and adult RA [7].

It is structurally composed of a sulfonamide (sulfapyridine) and a 5-aminosalacyclic acid (5-ASA) linked by an azo bond. Sulfasalazine inhibits GSH-H-transferase, the activation of NF-κB and, the formation of TNFα [8,9]. It is generally considered as a safe medication, but several cases of renal injury have been reported [10,11,12,13]. However, in long-term use and at high doses sulfasalazine is associated with adverse effects and toxicity due to its non-selective distribution and lack of specificity towards rheumatic organs/tissues representing a major concern for its clinical use.

On the other hand, in RA inflammation of the synovial tissue is the main characteristic at a cellular level since the synovium becomes infiltrated with T cells, dendritic cells, macrophages, fibroblasts, mast cells, neutrophils and B cells. Inflammatory mediators activate the formation of fibroblasts, increasing the production of connective tissue and consequently the formation of the pannus synovial. Pro-inflammatory mediators such as tumor necrosis factor alpha (TNF-α) are secreted by synovial macrophages and fibroblasts thereby stimulating T effector cells. TNF-α stimulates the production of other inflammatory mediators, such as interleukin 1 (IL-1), interleukin 6 (IL-6) and granulocyte-macrophage colony-stimulating factor, having a critically important function in regulating the balance between bone formation and destruction [14].

Direct intraarticular administration of new drug delivery systems capable of targeting SSZ to cell macrophages could be an interesting approach for avoiding the off-target toxicity of DMARDs agents, also preventing drug leaking from the synovial cavity. In this regard, drug-loaded PLGA microparticles are one of the candidates to be captured by macrophages; taking also into consideration that PLGA (poly lactic-co-glycolic acid) is a biodegradable and biocompatible polymer [15]. However, when macrophages encounter invaders, as in the case of microparticles, they generate an inflammatory response to eliminate them. Hence, silent nature towered macrophages cell functions are required for efficient drug carrier particles. If efficient particle uptake could be achieved without triggering undesirable responses, polymeric microparticles could be used as effective drug carriers. PLGA particles present such desirable silent nature regarding inflammatory responses [16]. In addition, it is known that passive targeting of microparticles to macrophages depends on several physical-chemical properties such as particle size, surface and composition, which must be taken into account in order to be rapidly phagocytosed [17,18]. In this sense, α-tocopherol could be used to modify the surface of the microparticles and to increase their lipophilicity, without increasing the inflammatory response of macrophages.

Therefore, the innovative concept of this work is the development and characterization of a new drug delivery system consisting of SSZ-loaded PLGA microparticles, prepared without and with the incorporation of α-tocopherol, able to directly targeting and being phagocytosed by cell macrophages, in order to increase the residence time of the drug in the intra-articular cavity, thereby increasing its efficacy and reducing the adverse effects which are associated to its non-selective biodistribution.

## 2. Materials and Methods

### 2.1. Materials

Sulfasalazine (SSZ) was purchased from Santa Cruz Biotechnology^®^ (Dallas, TX, USA). PLGA Resomer^®^ RG502 with a ratio of 50:50 poly(lactic-*co*-glycolic acid) was obtained from Evonik^®^ (Darmstadt, Germany). Polyvinyl alcohol (PVA, Mw 30,000–70,000 Da), fluorescein-5-isothiocyanate (FITC), dimethyl sulfoxide (DMSO) and α-tocopherol were supplied by Sigma-Aldrich^®^ (Darmstadt, Germany). Dichloromethane (DCM) was purchased from Thermo Fisher Scientific^®^ (Abingdon, UK). Buffers and solutions were prepared with distilled and deionized water obtained from Q-POD^®^ Milli-Q equipment (Merck Millipore, Burlington, MA, USA). 3-(4,5-dimethylthiazol-2-yl)-2,5-diphenyltetrazolium bromide (MTT) was supplied by Acros^®^. RAW 264.7 mouse macrophage cell line was obtained from ATCC^®^ TIB-71TM, (ATCC Company, Manassas, VA, USA). Mouse interleukin TNF-α, IL-1 and IL-6 ELISA kit were obtained from Cusabio^®^ (Houston, TX, USA). RevertAid H Minus First Strand cDNA Synthesis kit was supplied by Thermo Fisher Scientific^®^ (Abingdon, UK) and GeneAmp^®^ PCR System 9700 thermocycler was obtained from Applied Biosystems^®^ (Waltham, MA, USA).

### 2.2. Preparation of Microparticles

Sulfasalazine microparticles were prepared by the solvent extraction–evaporation method from an O/W emulsion. The aqueous phase consisted of 20 mL of 0.5% PVA solution, and the organic phase was prepared by dissolving 200 mg of PLGA 502 in 4 mL DCM, and then adding SSZ with or without α-tocopherol. The emulsion was formed by adding dropwise with a 25 G syringe the organic phase over the aqueous phase and using a Kinematica Polytron™ PT 10/35GT homogenizer (Fisher Scientific^®^, Waltham, MA, USA) at 8500 rpm for 5 min. The emulsion was maintained in continuous stirring for 3 h at room temperature to completely remove the organic solvent. Matured MPs were then vacuum filtered, washed with distilled water and freeze-dried for 24 h (Lyo Quest^®^, Telsta Technologies S.L, Barcelona, Spain). Blank MPs without and with α-tocopherol were also prepared (formulations F-A0 and F-A0E) (Table 1). All these formulations (Table 1, formulations F-AS, F-ASE, F-A0 and F-A0E) were used to evaluate the anti-inflammatory activity.

Fluorescein-5-isothiocyanate (FITC) MPs without and with α-tocopherol were also prepared by the same procedure (Table 1, F-AF and F-AFE) and used to perform the uptake studies in macrophages. All formulations were prepared in triplicate.

### 2.3. Characterization of Microparticles

#### 2.3.1. Morphological Characterization and Size Distribution of Microparticles

The morphology of MPs was analyzed by scanning electron microscopy (SEM; JEM 6335F; JEOL, Tokio, Japan) at 20 KV. For this, samples were coated with a thin layer of colloidal gold in a cathodic vacuum evaporator. Laser diffraction analysis (Microtrac^®^ 3500, Largo, FL, USA) was used to determine the mean diameter and size distribution of the MPs. Mean diameter was expressed as volume diameter and size distribution was represented by volume-distribution graphic curves.

#### 2.3.2. Encapsulation Efficiency and Drug Loading

Encapsulation efficiency (EE%) was calculated as the ratio between the amount of drug content into the MPs and the amount of drug used for their preparation, by means of the following equation:

(1) $$\mathrm{EE}\%=\frac{\mathrm{amount}\;\mathrm{of}\;\mathrm{encapsulated}\;\mathrm{SSZ}\;\left(\mathrm{mg}\right)}{\mathrm{amount}\;\mathrm{of}\;\mathrm{initial}\;\mathrm{SSZ}\;\mathrm{used}\;\mathrm{in}\;\mathrm{the}\;\mathrm{elaboration}\;\mathrm{of}\;\mathrm{MPs}\;\left(\mathrm{mg}\right)}\times 100$$

Drug loading (DL) was expressed as the amount of SSZ in 100 mg of MPs, according to the following equation:

(2) $$\mathrm{DL}=\frac{\mathrm{amount}\;\mathrm{of}\;\mathrm{encapsulated}\;\mathrm{SSZ}\;\left(\mathrm{mg}\right)}{100\;\mathrm{mg}\;\mathrm{MPs}}$$

EE and DL were calculated for all the formulations.

SSZ was analyzed by the HPLC method described by Kabir et al. [19], which was adapted to our experimental conditions. For this, an exact amount of MPs (10 mg) was dissolved in DCM (1 mL), and the polymer was precipitated with ethanol (14 mL). This suspension was then centrifuged at 13,000 rpm for 5 min and the supernatant was removed and filtered through 0.45 μm filters.

The HPLC apparatus consisted of an HPLC Perkin Elmer chromatographic system (Waltham, MA, USA), an Emplower CromaNec XP v.1.0.4 software and a Perkin Elmer diode array detector 235C (Waltham, MA, USA). The mobile phase was composed of acetonitrile and phosphate buffer at pH 3.5 (40 mM) (20:80 *v*/*v*). Before use the mobile phase was degassed and filtered through 0.45 μm nylon membrane filters. Flow rate was set at 1 mL/min; the injection volume was 20 μL, a Mediterranea Sea C18 column was used (250 mm × 4 mm, 5 µm particle size; Teknokroma^®^, Madrid, Spain) and the wavelength was set at 360 nm. All analyses were performed at 25 ± 0.5 °C. The HPLC method was validated to demonstrate the absence of interference between SSZ and PLGA or α-tocopherol. The method was linear within the concentration range of 4.0–10.0 μg/mL, with a limit of detection (LOD) of 0.492 μg/mL and limit of quantification (LOQ) of 1.489 μg/mL.

#### 2.3.3. In Vitro Release Studies

In vitro release studies were carried out in a Memmert WNB 45 water bath (Memmert, Schwabach, Germany) at 37 ± 1 °C and 100 agitations/min. For this, 10 mg of SSZ-loaded PLGA MPs were suspended in 3 mL PBS at pH 7.4. At predetermined times, the samples were centrifuged at 3000 rpm for 5 min and the supernatant removed with a 30 G needle attached to a syringe in order to avoid removing the MPs. Then, the supernatant was filtered through 0.45 μm filters, the medium was replaced with 3 mL of fresh PBS at pH 7.4, and the MPs were resuspended. SSZ was quantified by either spectrophotometry at 359 nm or HPLC as required by the method sensitivity. Release tests were carried out in triplicate.

### 2.4. Cell Culture Studies

Cell culture studies were performed in a RAW 264.7 mouse macrophage cell line. Macrophages were cultured in RPMI medium 1640 (Lonza^®^, Walkersville, MD, USA) supplemented with 10% bovine fetal serum and 1% gentamicin at 37 °C and 5% CO_2_. Cell cultures were used to evaluate the uptake of MPs by macrophages and to study the effect of MPs on cell viability and on the anti-inflammatory response.

#### 2.4.1. Phagocytosis Studies

The uptake of MPs by macrophages was studied using two fluorescent formulations, which contained FITC or FITC and α-tocopherol (F-AF and F-AFE). For this, MPs were incubated for 0.5, 1, 2, 3 and 5 h with the RAW 264.7 mouse macrophage cell line. MW6 plates at a concentration of 2 × 10^6^ cells per well were used for each formulation. In each well, a round coverslip was placed on which the macrophages adhere. After 24 h, the medium was removed and replaced with fresh RPMI supplemented medium in which the MPs were maintained in suspension at a concentration of 0.8 mg/mL. After incubation, the supplemented medium was carefully removed and each well washed with PBS at pH 7.4 (1 mL). Afterwards, macrophages were fixed with methanol (1 mL) at −20 °C for 15 min. Then, the methanol content was removed, and the coverslip was kept in PBS pH 7.4 (1 mL) at 4 °C. After obtaining all the coverslips, the samples from each formulation were prepared by depositing a drop of the mounting DAPI medium (Sigma-Aldrich^®^, Darmstadt, Germany). Samples were analyzed by fluorescence microscopy (Nikkon^®^ NIS, B-2A filter and Shutter filter, Tokio, Japan) in the UV range.

#### 2.4.2. MTT Cell Viability Assay

For the MTT assay the RAW 264.7 mouse macrophage cell line was used with a concentration of 0.3 × 10^6^ cells per well seeded in supplemented RPMI medium (2 mL) in MW6-plates. After 24 h incubation the medium was replaced and cells were treated with the MPs (formulations F-A0, F-AS, F-AS0 and F-ASE). Formulations F-A0 and F-AS were assayed at a concentration of 1.4 mg/mL and formulations F-A0E and F-ASE at 1.6 mg/mL. These concentrations were selected to resemble the conditions used in the anti-inflammatory tests. Moreover, a blank solution, a negative control (dead macrophages), a positive control (live macrophages without treatment) and a SSZ solution at a concentration of 42 µg/mL (concentration obtained from the in vitro release of SSZ from the MPs) were also analyzed. After incubation for 24 h cell macrophages were centrifuged at 1000 rmp for 5 min, the supernatant was removed and the macrophages resuspended in 0.1 mL of MTT solution at a concentration of 0.5 mg/mL. Then, incubation was performed in 96-well plates for 4 h at 37 °C and 5% CO_2_. In this assay MTT is reduced to an insoluble dark-blue formazan crystal due to active mitochondria of live cells, whereas inactivated mitochondria of dead cells are not able to reduce MTT. Finally, formazan crystals were dissolved in 0.1 mL DMSO. Then, absorbances are measured at 595 nm by iMark™ microplate absorbance reader (Bio-Rad laboratories Inc., Hercules, CA, USA). Cell survival is calculated for the treated macrophages taking as reference the untreated cells (Mock), which are assigned the value “1”. This assay was carried out in triplicate.

#### 2.4.3. Anti-Inflammatory Activity of the MPs Formulations in Cell Cultures

Anti-inflammatory activity was determined by quantifying the cytokines interleukins; IL-1, IL-6 and TNF-α by means of ELISA. Firstly, and to reproduce an inflammatory reaction, cell cultures were treated with and without lipopolysaccharide (LPS), with the concentration of inflammatory mediators being then analyzed.

The anti-inflammatory effect of the MPs (formulations F-A0, F-A0E, F-AS and F-ASE) and SSZ-solution was determined by quantifying the cytokines IL-1, IL-6 and TNF-α. For this, macrophages were cultured in RPMI medium alone and with the incorporation of LPS (0.7 µg) to stimulate the inflammatory response. T-25 flasks were used with a concentration of 2 × 10^6^ cells per flask. Incubation times were 24 h and 72 h. Macrophages incubated with LPS but without any other treatment (Mock) were used as control cells. For all the formulations assayed, the amount of SSZ incorporated was 294 µg in 7 mL (42 µg/mL), which corresponds to the average amount of SSZ released from the SSZ-loaded PLGA MPs at 72 h. This amount corresponds to 1.4 mg/mL of SSZ for formulation F-AS and 1.6 mg/mL for the other formulations. After 24 h, the RPMI medium was removed, centrifuged at 3500 rpm for 5 min, and the supernatant was removed and stored at 4 °C. The remaining non-phagocytized MPs were resuspended in fresh RPMI medium. The same procedure was performed at time 72 h. The supernatants obtained were analyzed by ELISA, according to manufacturer’s instructions (mouse TNF-α ELISA kit, mouse IL-1 ELISA kit and mouse IL-6 ELISA kit, CUSABIO, USA) to determine the concentration of the inflammatory mediators, with macrophages being used to evaluate the gene expression.

#### 2.4.4. RNA Extraction

Gene expression of IL-1, IL-6 and TNF-α was determined by Retrotranscription-PCR (RT-PCR) using the macrophage culture obtained as indicated in Section 2.4.2 at 72 h. Macrophages were centrifuged at 1500 rpm for 10 min and resuspended in 1.0 mL Trizol^®^ Reagent (Ambion, Life Technologies, Carlsbad, CA, USA) for RNA isolation following manufacturer’s instructions. RNA concentration was measured using a GE NanoVue spectrophotometer (GE Healthcare Life Sciences, Hatfield, UK).

In order to determine the purity of the RNA extracted, RNA absorbance was measured at 260 nm and 280 nm for Mock, F-A0, F-A0E, F-AS, F-ASE and SSZ-solution. A260/A280 ratios were near 2 for all the formulations. The values obtained were in the range of 1.8–2.1, thereby indicating high RNA purity [20].

#### 2.4.5. Reverse Transcription-Polymerase Chain Reaction

A reverse transcription reaction was performed from 1 μg of extracted RNA using the Thermo Scientific RevertAid H Minus First Strand Cdna Synthesis kit (Thermo Fisher Scientific, Waltham, MA, USA), according to the manufacturer’s instructions. Then, the obtained cDNA was used in a conventional PCR reaction designed to amplify specific fragments of the selected protein effector genes. The specific forward and reverse primers used are shown in Table 2. The PCR was performed in a total volume of 50 μL, using 1X Phusion Flash High-Fidelity PCR Master Mix (Thermo Fisher Scientific, Waltham, MA, USA), 0.2 μM of the specific primers and 2 μL of cDNA.

The PCR reaction was carried out using a GeneAmp^®^ PCR system 9700 thermocycler (Applied Biosystems, Foster City, CA, USA). The amplification parameters were: 3 min at 95 °C; 40 cycles of 1 min at 95 °C, 1 min at 55 °C and 2 min at 72 °C; with a final extension step of 3 min at 72 °C. The amplified samples were visualized after electrophoresis in a 2% agarose gel. The visualized fragments were quantified by densitometry using the Image J software (National Institutes of Health, Bethesda, MD, USA). Additionally, RT-PCR analysis was performed to amplify a constitutive gene from mice (glyceraldehyde-3-phosphate dehydrogenase (GADPH)) in order to assure that these extracted RNA samples contained approximately the same number of cells and thus standardize RNA concentration for these comparative analyses between in vitro models. Specific primers designed for GADPH, IL-6, IL-1 and TNF-α are showed in Table 2.

Mock band (macrophages incubated with LPS but without any other treatment) were used as control cells and assigned a value of 1. The intensity of the other bands was calculated as a function of this reference value. Additionally, a negative control (NC) was used to determine if the materials and methodology employed exert an influence on the results obtained.

#### 2.4.6. Statistical Analysis

Data were analyzed using Statgraphics version 18 × 64-bit (Statgraphics Technologies, Inc., The Plains, VA, USA). Data were expressed as mean ± standard error of the mean of three independent experiments and analyzed by one-way analysis of variance (ANOVA).

## 3. Results and Discussion

Intraarticular infiltrations are common practice in osteoarthritis and rheumatoid arthritis. In rheumatoid arthritis, macrophages are the target cells for the uptake of therapeutic drug-containing particles since they constitute the local and systemic amplifiers for inflammation [21]. Macrophages are innate immune cells of the mononuclear phagocyte system playing an important role in inflammatory response.

It is known that passive targeting to macrophages requires a combination of physical properties, such as particle size and shape [17]. Passive targeting involves the optimization of the physicochemical characteristics of MPs (composition, size, shape, surface chemistry, elasticity or rigidity) to obtain increased uptake by macrophages [17,18,22]. Additionally, macrophages are capable of ingesting particles having a diameter between 1–10 µm in order to eliminate them from the body [23]. The surface charge of particles is thought to be critical for endocytic uptake, as the incorporation of lipophilic substances increases the uptake of MPs by macrophages [24]. Recently, α-tocopherol has been used in the preparation of 5-fluorouracil nanoparticles destined to treat oral squamous cell carcinoma [25]. Gao et al. [26] developed hyaluronic acid-tocopherol succinate-based self-assembling micelles for delivering rifampicin to alveolar macrophages. However, the effect of incorporating α-tocopherol in the uptake of PLGA MPs by macrophages has not yet been studied. For this, in this work a new delivery system was developed consisting of SSZ-loaded PLGA MPs without and with the incorporation of α-tocopherol. The formulation is intended for intraarticular administration and to specifically target macrophages in order to directly act on the inflammatory response.

**Figure 1:**
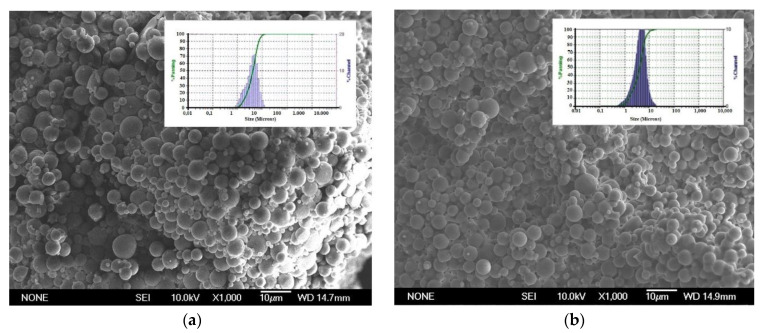

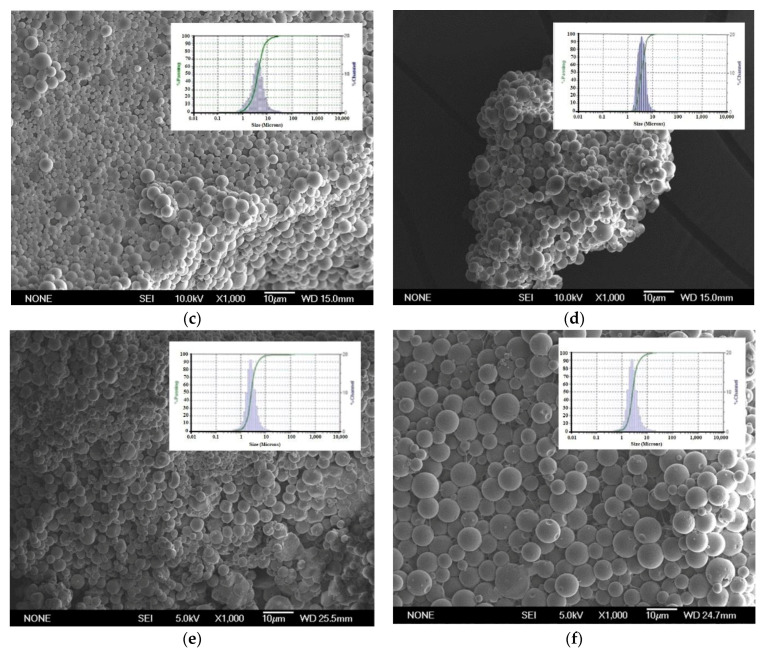
SEM images and particle size distribution corresponding to formulations (**a**) SSZ-loaded MPs, (**b**) SSZ-loaded MPs with α-tocopherol, (**c**) blank MPs, (**d**) blank MPs with α-tocopherol, (**e**) FICT-loaded MPs and (**f**) FICT-loaded MPs with α-tocopherol. SSZ: sulfasalazine, FICT: fuorescein-5-isothiocyanate.

SSZ-loaded PLGA MPs without and with α-tocopherol (Table 1, formulations F-AS and F-ASE) were obtained by the solvent extraction evaporation method. SEM analysis revealed that particles from both formulations are spherical with similar shapes and smooth surfaces (Figure 1a,b). Mean particle size was 4.09 ± 0.64 μm and 3.87 ± 0.21 μm for formulations F-AS and F-ASE, respectively (Table 3) being adequate for macrophage uptake [27]. MPs from both formulations also present narrow particle size distributions as seen in Figure 1. No significant differences were found between loaded and non-loaded MPs (Figure 1).

Table 3 shows the results of drug loading (DL) and encapsulation efficiency (EE%) for both SSZ formulations (F-AS and F-ASE). High values of these two parameters are desirable in order to reduce the dose of MPs given by intraarticular administration. Mean values of EE for SSZ in formulations F-AS and F-ASE were 63.50 ± 6.62% and 81.07 ± 11.92%, respectively. The difference found could be due to the fact that α-tocopherol increases the liposolubility of the polymeric matrix, thereby increasing the solubility of SSZ in the matrix during the encapsulation process. SSZ has low aqueous solubility (13 mg/L at 25 °C). The incorporation of α-tocopherol when preparing the MPs did not have any influence on DL.

**Figure 2:**
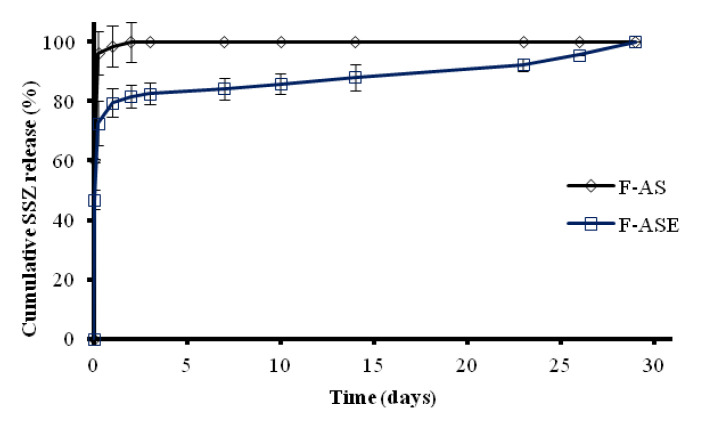
Mean release profiles (± SEM) of SSZ from F-AS and F-ASE microparticle formulations. SSZ: sulfasalazine.

When performing in vitro release tests at pH 7.4, formulation F-AS exhibited a very rapid release with complete release of SSZ after 3 days (Figure 2). At this time formulation F-ASE released around 80% of the drug content. After this initial phase slow SSZ release occurred for 20 days. It has been described that the incorporation of α–tocopherol when preparing PLGA MPs can either increase or decrease the release rate of encapsulated drugs [28,29]. In our case, the incorporation of α–tocopherol allowed for controlling the release of the drug, which is desirable for prolonging its action.

After verifying that α-tocopherol increased the encapsulation of SSZ within PLGA MPs and better control of drug release was achieved, the effect of this surface modifier was evaluated in the uptake of PLGA MPs by macrophages. This effect has not been previously studied. As SSZ is not a fluorescent agent, for this we have studied this effect using FITC-loaded PLGA MPs prepared without and with α-tocopherol (Table 1, formulations F-AF and F-AFE). FITC is a fluorescent dye thereby allowing for the detection of MPs captured by cell macrophages.

SEM analysis revealed that FITC-loaded PLGA MPs have spherical shapes and smooth surfaces (Figure 1e,f). Mean particle sizes are 3.98 ± 0.91 μm and 5.36 ± 0.62 μm for F-AF and F-AFE, respectively (Table 2). These particle sizes are similar to those of SSZ MPs being both adequate for macrophage uptake [27,30]. Additionally, particle sizes exhibited narrow distributions.

**Figure 3:**
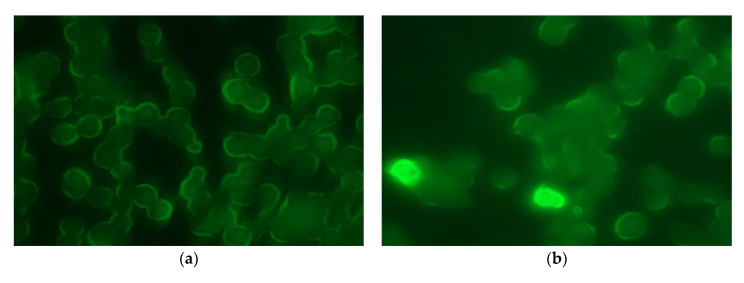

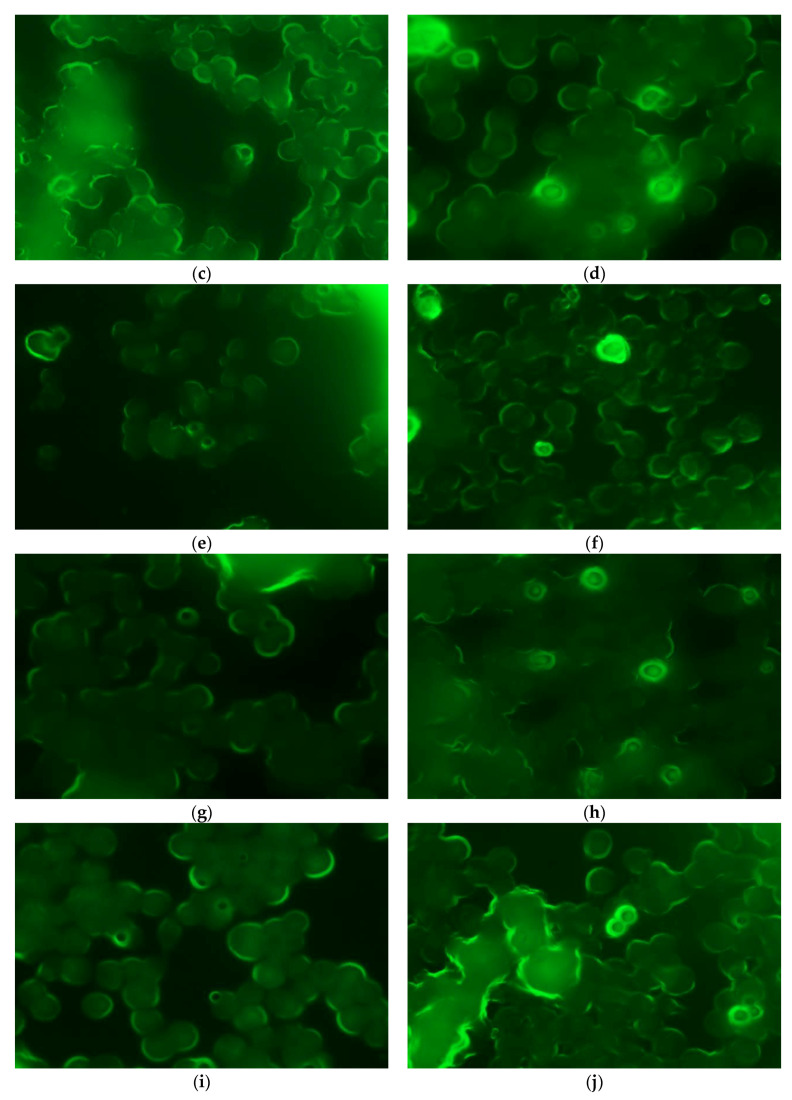
Fluorescence microscopy images (×60) of macrophages obtained at different times after incubation with formulations: (**a**) F-AF at 0.5 h, (**b**) F-AFE at 0.5 h, (**c**) F-AF at 1 h, (**d**) F-AFE at 1 h, (**e**) F-AF at 2 h, (**f**) FAFE at 2 h (**g**) F-AF at 3 h, (**h**) F-AFE at 3 h, (**i**) F-AF at 5 h and (**j**) F-AFE at 5 h.

Figure 3 shows fluorescence microscopy images obtained for both formulations at different incubation times (0.5, 1, 2, 3 and 5 h). In all images macrophages showed autofluorescence due to the heterogeneous nature of the different cell components, such as NAD(P)H, flavins, porphyrin and lipofuscin [31,32]. After 30 min MPs from formulation F-AFE were observed inside the macrophages (Figure 3b), with the same occurring after 1 h for formulation F-AF (Figure 3c) thereby indicating that the incorporation of α-tocopherol improves the uptake of MPs by macrophages.

Mathaes et al. [24] indicated that particles with lipophilic surfaces are more readily phagocytized than those more hydrophilic. In our case, the presence of lipophilic α-tocopherol could have modified the surface of MPs leading to better access inside cell macrophages. Once uptake by macrophages took place MPs remained inside for at least 5 h (Figure 3i,j). Moreover, incubation of formulation F-AF resulted in the observance of dark fluorescence spots inside the cells probably due to the rapid diffusion of FITC outside the MPs (Figure 3c,e,g,i). On the contrary, formulation F-AFE prepared with α-tocopherol showed the fluorescent dye FITC inside the polymeric matrix (Figure 3b,d,f,h,j), which could be attributed to the fact that α-tocopherol increases the lipophilicity of the polymeric matrix of MPs thereby decreasing the release of FITC.

**Figure 4:**
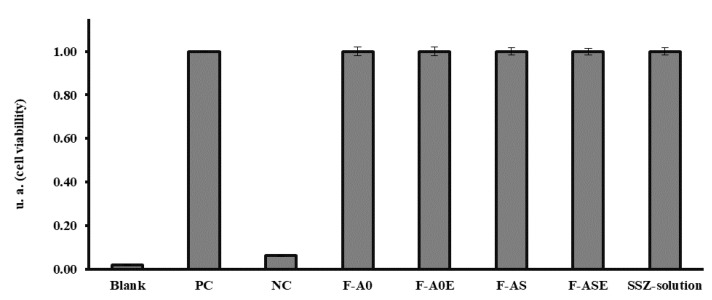
Cell viability results obtained after incubation of macrophages with formulations F-A0, F-A0E, F-AS, F-ASE and SSZ-solution. PC: positive control (live macrophages) and NC: negative control (dead macrophages).

Evaluation of cytotoxicity is important when developing new drug delivery systems as several factors such as composition, size and surface characteristics of the system may have an influence on cell viability. For this reason, the possible cytotoxic effect of blank PLGA MPs (F-A0 and F-A0E), SSZ-loaded PLGA MPs (F-AS and F-ASE) and SSZ solution were studied in RAW 264.7 macrophages by the MTT assay after 24 h incubation. The results are shown in Figure 4, where it can be seen that macrophages treated with the different formulations exhibited similar cell viability values than non-treated cells (PC cells).

Prior to study if the incorporation of α-tocopherol in the preparation of SSZ-loaded MPs has an influence on the anti-inflammatory response, the effect of LPS on the inflammatory response was analyzed. The inflammatory mediators involved in the pathology of Rheumatoid arthritis are the cytokines TNF-α and IL-6 which play a role in the local activity of the disease, inducing synovitis with IL-1 destroying cartilage. In addition, TNF-α and IL-1 regulate IL-18 involved in the expression of IFN-γ [33,34]. In our case the results obtained showed significant differences in the concentrations of IL-1, IL-6 and TNF-α when LPS was included in the culture medium (Table 4).

When comparing the influence of LPS in control and formulations F-A0 and F-A0E (non-loaded MPs) in the production of IL-1, IL-6 and TNF-α by two-way ANOVA, statistically significant differences were found due to the presence of LPS. In all cases *p* < 0.001. Exposure to LPS led to significant increases in the production of IL-1, IL-6 and TNF-α of up to 1.3, 1.3 and 2.1 times and 1.5, 1.7 and 2 times for formulations F-A0 and F-A0E, respectively, as compared to their respective control groups. These results are in agreement with the fact that LPS, which derives from Gram-negative bacteria cell walls [35] is able to induce in macrophages the production of proinflammatory cytokines such as TNF-α and interleukins IL-1 and IL-6 [36,37], which contributes to the progression of various inflammatory diseases [36,37,38]. For this, in our study macrophages were cultured in RPMI medium supplemented with LPS to stimulate the inflammatory response.

On the other hand, when comparing control and formulations F-A0 and F-A0E, statistically significant differences are only found regarding TNF-α levels (*p* < 0.001) but not for IL-1 levels (*p* = 0.063) and IL-6 levels (*p* = 0.406).

**Figure 5:**
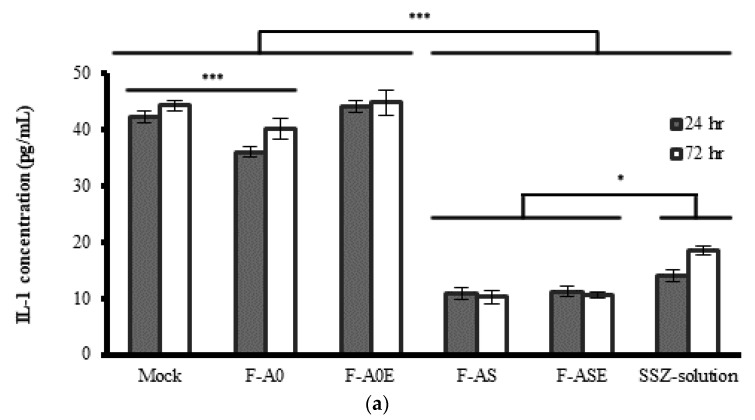

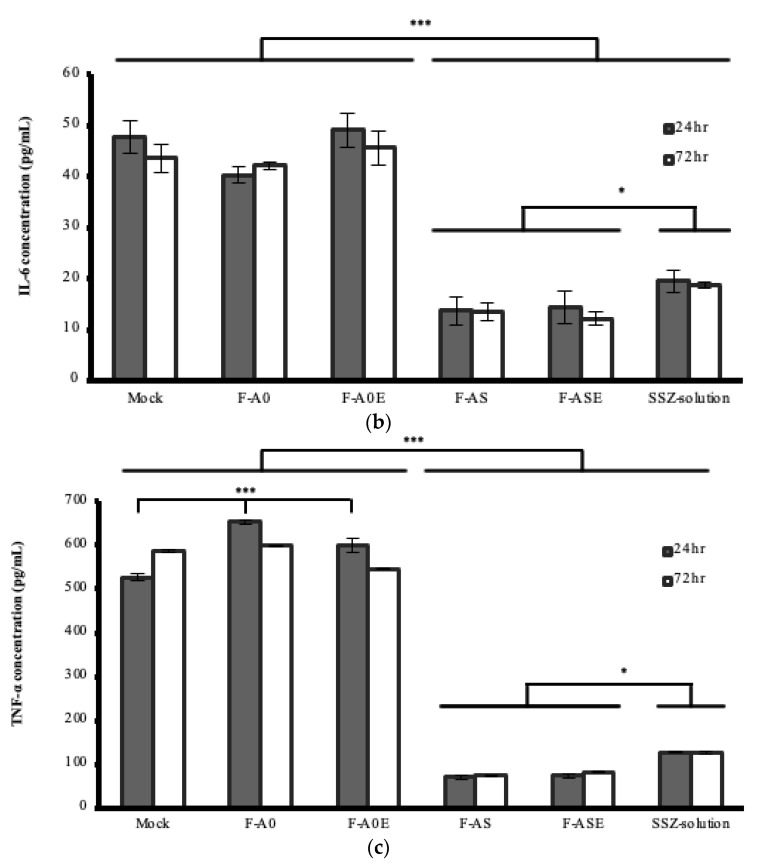
Concentrations of (**a**) IL-1, (**b**) IL-6 and (**c**) TNF-α obtained after incubating macrophages with formulations F-A0, F-A0E, F-AS, F-ASE and SSZ-solution for 24 h and 72 h. Mock: control. *** *p* < 0.001; * *p* < 0.05. See text for details.

The anti-inflammatory response of SSZ solution, SSZ-loaded PLGA MPs prepared without and with the incorporation of α-tocopherol (F-AS and F-ASE) and blank PLGA MPs (F-A0 and F-A0E) was studied in macrophages by determining the concentrations of interleukins IL-1, IL-6 and TNF-α at 24 h and 72 h. Figure 5 shows the results obtained. To evaluate the results obtained, in a first step a two-way ANOVA analysis was used for comparing control and non-loaded MPs. In a second step, a two-way ANOVA was used for comparing all SSZ-containing formulations being the variables compared incubation time and formulation.

In all cases, the concentrations of inflammatory mediators were high as the presence of LPS stimulates their production. The concentration of IL-1 obtained at 24 h and 72 h was significantly reduced in the presence of blank MPs (*p* < 0.001) but not affected by the incorporation of α-tocopherol in blank MPs (Figure 5a). It has been indicated that α-tocopherol exhibits anti-inflammatory effects both in vitro and in vivo, being able to decrease pro-inflammatory cytokines at high doses [39]. In our case, this effect was not observed, probably due to the fact that the amount of α-tocopherol incorporated in the preparation of the formulations was very low.

All SSZ-containing formulations (F-AS, F-ASE, SSZ-solution) produced a statistically significant reduction in IL-1 concentrations after 24 h and 72 h when compared to control/blank formulations (*p* < 0.001). When SSZ was encapsulated within MPs the average reduction of IL-1 concentration ranged between 69.9% and 76.3% at both incubation times without significant differences found between both formulations. However, in the second step when a two-way ANOVA was carried out for the SSZ-containing formulations, a significant increase of the IL-1 levels occurred for SSZ-solution (*p* = 0.032), indicating a decrease of the anti-inflammatory response produced by this formulation.

When blank MPs were prepared with the incorporation of α-tocopherol, the concentration of IL-6 was non-significantly affected (*p* > 0.05) (Figure 5b). As occurred with IL-1, all formulations including SSZ produced a significant reduction in IL-6 concentrations at both incubation times (*p* < 0.001). When comparing all SSZ-containing formulations significant differences were found between SSZ-loaded MPs and SSZ-solution (*p* = 0.040).

Blank MPs (F-A0 and F-A0E) produced a significant increase in the concentration of TNF-α at 24 h (*p* < 0.001) (Figure 5c). All formulations including SSZ resulted in a significant reduction of TNF-α at both times (*p* < 0.001), with the highest reduction (85–89%) obtained with formulations F-AS and F-ASE, respectively when compared to blank formulations.

The results obtained indicate that SSZ-loaded PLGA MPs prepared without and with the incorporation of α-tocopherol were able to suppress the expression of the inflammatory mediators IL-1, IL-6 and TNF-α when compared to SSZ in solution, thereby indicating that the release of SSZ from PLGA MPs is capable of producing a significant inhibition of the inflammatory response induced by LPS. These results could be explained by the fact that MPs captured by macrophages are able to release SSZ at its site of action.

**Figure 6:**
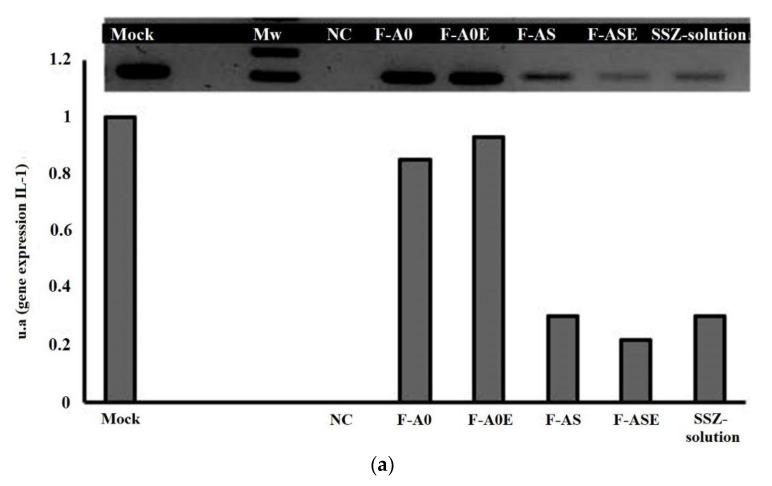

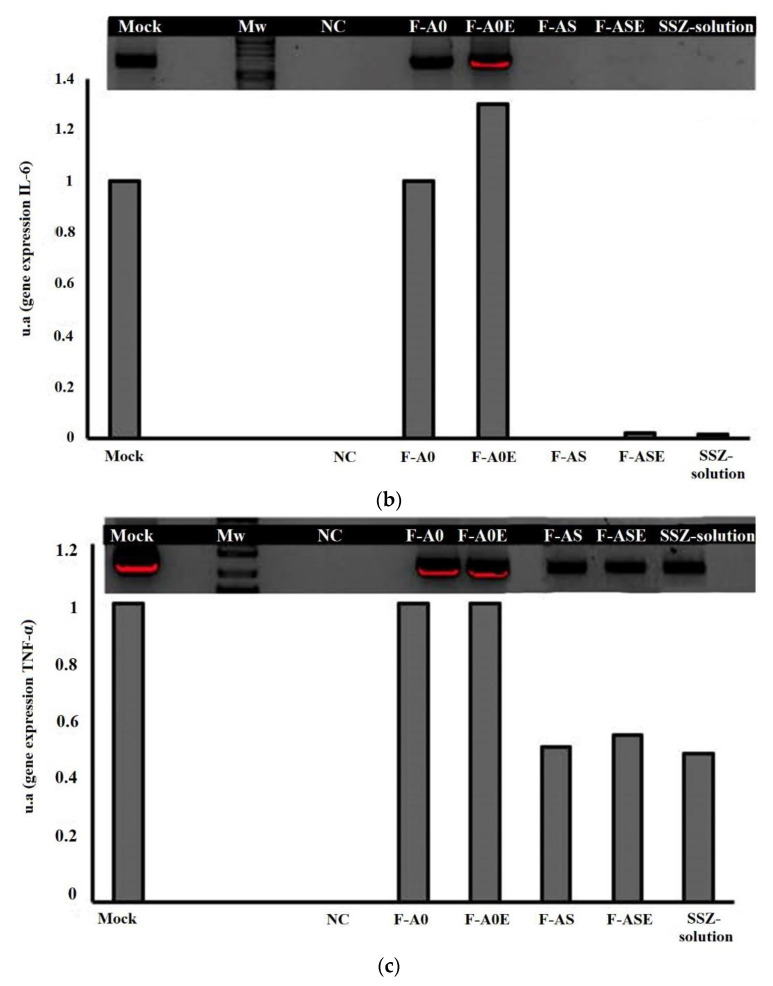
Agarose gel electrophoresis of PCR products and mRNA expression levels of (**a**) IL-1, (**b**) IL-6 and (**c**) TNF-α obtained after 72 h incubation with formulations F-A0, F-A0E, F-AS, F-ASE and SSZ-solution. Mock: control, NC: negative control, Mw: molecular-weight size marker.

The effect of SZZ formulations on gene expression of these cytokines was evaluated by RT-PCR analysis (Figure 6). According to our results, gene expression of IL-1 was very slightly reduced by blank MPs (formulations F-A0 and F-A0E). However, a marked decrease of gene expression was observed for all SSZ-containing formulations (F-AS, F-ASE and SSZ-solution). The best results correspond to SSZ-loaded PLGA MPs prepared with α-tocopherol (Figure 6a). For this formulation, the reduction was close to 70%. The results obtained for IL-6 gene expression showed that the presence of blank MPs did not affect gene expression, whereas blank MPs prepared with α-tocopherol resulted in a slight increase (Figure 6b). An almost complete inhibition of IL-6 gene expression occurred with all formulations incorporating SSZ either encapsulated or in solution. Regarding TNF-α gene expression, the presence of blank MPs did not have any effect (F-A0 and F-A0E). However, an almost 50% decrease was observed with all the formulations prepared with SSZ (Figure 6c).

The inhibitory effect of these inflammatory mediators, observed with the SSZ formulations, occurs at the gene expression level, suggesting that SSZ may exhibit either a direct mechanism of action on them or act inhibiting the factors involved in the transcription phenomena of these cytokines, like NF-κB, which is involved in the activation of pro-inflammatory mediators such as IL-1β, TNF-α and IL-6. This transcription factor is a key regulator of both contraction-associated genes and pro-inflammatory cytokines [40].

Taking into account the rapid elimination of SSZ when give in solution, which hinders its permanence inside the synovial cavity, the use of microparticulate systems will allow increasing the residence time of SSZ, due to the uptake of SSZ-loaded PLGA MPs by synovial macrophages and, the adequate controlled release obtained for SSZ when the MPs are prepared with α-tocopherol, which is desirable for prolonging its duration of action.

## 4. Conclusions

The new formulation developed for SSZ as PLGA MPs prepared with α-tocopherol produced a decrease in IL-1, IL-6 and TNF-α levels thereby resulting in an adequate delivery system for the treatment of chronic inflammatory diseases such as rheumatoid arthritis. This new delivery system will allow increasing the residence time of SSZ inside the synovial cavity as a consequence of its uptake by macrophages, decreasing the adverse effects associated with its non-selective biodistribution. Moreover, microparticles prepared with α-tocopherol will adequately control the release of SSZ, thereby prolonging its duration of its action. Therefore, this new delivery system consisting of SSZ-loaded-PLGA MPs prepared with α-tocopherol show promising results for further research.

## Figures and Tables

**Table 1:** Formulations developed. SSZ (sulfasalazine) and FITC (fluorecein-5 isothiocyanate).

Formulation	SSZ (%)	FITC (%)	α-tocopherol: Polymer
F-AS	5	-	-
F-ASE	5	-	1:5
F-A0	-	-	-
F-A0E	-	-	1:5
F-AF	-	10	-
F-AFE	-	10	1:5

**Table 2:** Forward and reverse primers used for RT-PCR.

Formulation	Forward	Reverse
GADPH	5′-TGAGGCCGGTGCTGAGTATGTCG-3′	5′-CCACAGTCTTCTGGGTGGCAGTG-3′
IL-1	5′-AGTTGACGGACCCCAAAAGAT-3′	5′-GTTGATGTGCTGCTGCGAGA-3′
IL-6	5′-CTTCCATCCAGTTGCCTTCTTG-3′	5′-AATTAAGCCTCCGACTTGTGAAG-3′
TNF-α	5′-GATCTCAAAGACAACCAACATGTG-3′	5′-CTCCAGCTGGAAGACTCCTCCCAG-3′

**Table 3:** Drug loading (DL), encapsulation efficiency (EE%) and mean particle size for formulations F-AS, F-ASE, F-AF and F-AFE.

Formulation	DL (mg of Compound/100 mg of MPs)		EE%	Mean Particle Size (µm)
	SSZ	FICT		
**F-AS**	3.02 ± 0.31	-	63.50 ± 6.62	4.09 ± 0.64
**F-ASE**	3.24 ± 0.48	-	81.07 ± 11.92	3.87 ± 0.21
**F-AF**	-	0.11 ± 0.02	1.22 ± 0.24	3.98 ± 0.91
**F-AFE**	-	0.09 ± 0.01	1.13 ± 0.16	5.36 ± 0.62

**Table 4:** Concentrations of IL-6, IL-1 and TNF-α obtained for control cells and formulations F-A0 and F-A0E (non-loaded MPs) before and after exposing cell macrophages to LPS (lipopolysaccharide).

Formulation	LPS (μg)	Concentration (pg/mL)		
		IL-1	IL-6	TNF-α
Control	-	22.6 ± 0.13	25.3 ± 0.03	220.6 ± 0.3
	0.7	43.27 ± 2.79	47.70 ± 10.46	526.08 ± 0.088
F-A0	-	28.9 ± 0.23	30.2 ± 0.03	308.9 ± 0.45
	0.7	36.38 ± 1.63	40.35 ± 1.63	653.38 ± 3.5
F-A0E	-	30.2 ± 0.02	29.83 ± 0.02	300.2 ± 0.23
	0.7	44.20 ± 4.24	49.23 ± 11.06	600.4 ± 14.13

## Data Availability

Not applicable.
